# Biofunctionalization of Silver Nanoparticles With Lactonase Leads to Altered Antimicrobial and Cytotoxic Properties

**DOI:** 10.3389/fmolb.2019.00063

**Published:** 2019-08-06

**Authors:** Kshitiz Gupta, Sanjay Chhibber

**Affiliations:** Department of Microbiology, Panjab University, Chandigarh, India

**Keywords:** silver nanoparticles, lactonase, antibiofilm activity, cell cytotoxicity, antivirulence

## Abstract

**Background:** N-acylated homoserine lactone lactonase which cleave the Acyl homoserine lactone molecules produced by biofilm-forming pathogens and silver nano-particles (AgNPs), are known for their antibacterial effect against several Gram-positive and Gram-negative bacteria. In this study, AgNPs were coated with N-acylated homoserine lactonase protein (AgNPs-AiiA) isolated from *Bacillus* sp. ZA12.

**Results:** The AgNPs-AiiA complex was characterized by UV-visible spectra, Dynamic light Scattering, Fourier transform infrared spectroscopy (FTIR), and Field Emission Scanning Electron Microscope (Fe-SEM). The synthesized nano-particles were found to be spherical in shape and had an approximate size of 22.4 nm. Treatment with AiiA coated AgNPs showed a significant reduction in exopolysaccharide production, metabolic activity, cell surface hydrophobicity of bacterial cells, and anti-biofilm activity against multidrug-resistant *K. pneumoniae* as compared to treatment with AiiA protein and neat AgNPs. AgNPs-AiiA complex exhibited potent antibiofilm activity at sub-optimal concentration of 14.4 μg/mL without being harmful to the macrophages and to the various tissues including kidney, liver, spleen and lungs of BALB/c mice upon intra-venous administration.

**Conclusion:** It is concluded that at a concentration of 14.4 μg/mL, AgNPs coated with AiiA kill bacteria without harming the host tissue and provides a suitable template to design novel anti-biofilm drug to circumvent the issue of drug resistance.

## Background

Antimicrobial resistance is on the increase and has become a global problem. This phenomenon is not new as within 20 years of its discovery, Alexander Fleming observed that microbes became “educated” to resist penicillin. The increase in drug resistance has primarily taken place due to their misuse and abuse (Składanowski et al., 2016). Gradually, the time from introduction of new class of drug to detection of resistance has been constantly decreasing (Ventola, 2015). In order to tackle this menace, the scientific community is looking for alternative therapies. Research investments on the development of novel agents that are effective against antimicrobial resistant microorganisms has raised interest in other non-conventional alternative therapies, including the use of bacteriophage, antimicrobial peptides, and quorum quenching nanomaterials (Doss et al., 2017; Ma et al., 2018).

It has been reported that between 65 and 80% of infections are associated with biofilm formation, either directly or via devices such as a catheter, ureteral and coronary stents, and interocular lens, etc. (Rémy et al., 2018). Biofilm formation is often induced via quorum sensing (QS) which consists of a heterogeneous structure, embedded in an extracellular matrix (ECM). The ECM is made of polysaccharides, proteins, and extracellular DNA that acts as a barrier for antibiotics inside cells, inducing antibiotic tolerance. The biofilm environment combines high bacterial cell density and high selection pressure, thus increasing the frequency of formation of resistant cells either through random mutations or gene transfer (Limoli et al., 2015). Such cells survive antibiotic treatment by being in a different physiological state at the time of treatment (Miyaue et al., 2018). They are 100–1,000 times more immune to antimicrobial therapy as compared to planktonic lifestyle (Singh S. et al., 2017). So, eliminating biofilms is a challenge in controlling and treating hospital acquired infections (HAIs). Since QS plays an important role in the homologous communication between prokaryotes and heterologous communication between prokaryote and eukaryotes, it makes sense that competitors must have evolved various mechanisms to silence the QS system of other bacteria. This ability to disrupt bacterial communication can be widely found in different kinds of organisms including bacteria (Dong and Zhang, 2005; Romero et al., 2015), marine algae (Givskov et al., 1996), terrestrial plants (Gao et al., 2003), and mammalian cells (Camps et al., 2011). Although quorum quenching (QQ) is a general term used to describe any form of QS interference, it was specifically coined to relate to the enzymatic quenching of AHL QS signals (Dong et al., 2001). Enzymatic QQ is classified in three main groups: lactonases, oxido-reductases, and acylases (Gohil et al., 2018). They mainly target AHLs and AI-2 mediated QS (Webster, 2012; Hauser et al., 2016).

Apart from quorum quenching therapy, nano-therapy is seen as a plausible replacement for antibiotic therapy (Hajipour et al., 2012). Based on several studies, it has been suggested that bacteria might not be able to develop antimicrobial resistance against metal nano-particles (NPs) (Dizaj et al., 2014; Ranoszek-Soliwoda et al., 2017). Owing to their small size, NPs can easily pass through cell barriers and result in disruption of cell. One such nanomaterial is silver nanoparticle (AgNP) whose antibacterial property has been reported since ancient times. It has wide applications in water and air purification, household products, cosmetics, biomedical products (Shenashen et al., 2014; Singh T. et al., 2017), food production (Zhang et al., 2018), and clothing (Iravani et al., 2014).

Considering various benefits, the antivirulent and antibacterial properties of lactonase (AiiA) and AgNPs, respectively, were combined to treat planktonic and biofilm cells of *K. pneumoniae* B5O55, *in vitro*. Since AgNPs are known to endow some cell toxicity *in vivo* when used in high doses, several modifications on its surface have been done to either increase their permeability or to increase their bio-compatibility (Das et al., 2017b). In one study, the addition of sodium borohydride to simultaneously reduce disulfide bonds of the derivatives and silver ions facilitated the reaction of thiol groups with newborn silver atoms to form nano-clusters. Such assemblies made the clusters bio-compatible and led to the better penetration of the NPs inside fibroblast cells (Wang et al., 2017). Similarly, functionalization of AgNPs with BSA and PEG also reduced the generation of NADPH mediated oxidative stress (Das et al., 2017b).

The cell toxicity of the combination therapy was checked on primary macrophage cell line by MTT assay so as to discern the plausibility of using this therapy *in vivo*. In addition, the toxicity of neat AgNPs (AgNPs formed through citrate reduction) and AgNP—AiiA complex (AgNPs formed by reduction of AgNO_3_ with AiiA) on various organs of BALB/C mice was compared after injecting the compounds intravenously into BALB/c mouse.

## Materials and Methods

### Ethics Statement

The experimental protocols were performed with prior approval from Institutional Animal Ethics Committee of Panjab University, Chandigarh, India (Approval ID: IAEC/156). All the experiments concerning animals was complied with the guidelines of Committee for the Purpose of Control and Supervision of Experiments on Animals (CPCSEA), Government of India.

### Bacterial Strain

The *K.pneumoniae* standard strain B5O55 (O1:K2, MTCC 5832, a prototrophic strain isolated by Dr. M. Trautman, Department of Microbiology and Hygiene, Ulm, Germany) was obtained. *Bacillus* sp. ZA12 (Accession number BU595363) was previously isolated in our laboratory from soil sample taken from parts of Chandigarh (India). This strain served as a source of lactonase enzyme (AiiA). The isolates were stored in 50% glycerol at −70°C. Stock cultures were used to inoculate Luria broth and incubated at 37°C and 30°C for *K. pneumoniae* and *Bacillus*, respectively.

### Standardization of AHL Lactonase Activity of AiiA in Different Physical Conditions

AiiA from soil isolate (*Bacillus* sp. Accession number BU595363) was previously cloned in *E. coli* DH10β and over expressed in *E. coli* BL21 by inducing with IPTG. It was purified by Ni-NTA (Nickel NTA) affinity chromatography and its molecular size was confirmed by running on SDS-PAGE (Manuscript Id: 239501, International Journal of Antimicrobial Agents). The purified protein (AiiA) was dissolved in 1× PBS buffer and stored at −80°C. pH and temperature conditions were varied between 5–8 and 25–50°C, respectively to find the optimum conditions at which activity of enzyme was highest (Additional File 1). These conditions were used for subsequent experiments. To calculate the rate of reaction, 500 μl of 100 μg/ml purified AiiA was incubated with 10 μM C8-HSL at 28°C. The reaction was stopped by adding 0.2% SDS after 1.5, 3, 5, 7 h. After the reaction was terminated, residual amount of AHL was estimated by well diffusion assay with the help of *C. violaceum* 026 (ATCC 12472) (Miller, 1972). *C. violaceum* is a bio-reporter strain that produces violet color upon contact with AHLs. A decrease in diameter of the zone of violet color was considered as the decrease in amount of AHLs. The diameter of the violet color zone was compared with the standard curve prepared by using known concentrations of AHL and a reduction in nM of AHL/minute/100 μg lactonase was calculated.

### Synthesis of Silver Nitrate Nanoparticles (AgNPs)

AgNPs were synthesized by chemical reduction method (Perumal and Mahmud, 2013). The concentration of AgNO_3_ solution was varied in the range of 0.0005 M- 0.01 M. To ensure the controlled growth of NPs, the solution of AgNPs was prepared in 100% ethanol. Silver ions were reduced by AiiA solution. For this AiiA solution, 100 mgL^–1^ was prepared in 1X PBS buffer (pH 7.2). AgNO_3_ solution in different concentrations was mixed with AiiA solution and the mixture was incubated under gentle shaking till a change in color from transparent to pale yellow was observed, indicating the reduction of silver ions to form metallic silver. After incubation, the mixture was centrifuged at 11,000 rpm for 40 min. and washed several times with de-ionized water. The formation of NPs was confirmed by aggregation method in the presence of NaCl solution. The size and zeta potential of NPs was determined by dynamic light scattering (DLS). The optical density of AgNPs was checked till 7 days at 435 nm to observe the stability and nucleation. AgNPs synthesized upon reduction with sodium citrate were used for comparison.

### Field Emission Scattering Electron Microscopy (Fe-SEM) and Transmission Electron Microscopy (TEM)

The morphological characteristics of AgNPs thus formed were determined through Field Emission Scanning Electron Microscope (Fe-SEM) and TEM. For Fe-SEM, the solution of AgNP was critically point dried, coated with platinum by using a fine coat ion sputter JFC-1100. Specimens were examined at varying voltages using Hitachi SU8010 FeSEM at SAIF (Sophisticated Analytical Instrumentation Facility), Panjab University, Chandigarh. TEM measurements were done with the help of HITACHI H-7500, operating at 100 kV with magnification of 400000 X. The TEM grid was prepared by placing a drop of diluted solution of AgNPs on a carbon-coated copper grid and later drying it under a lamp.

### Fourier Transform Infrared Spectroscopy (FTIR) of Adsorbed Protein

The infrared spectra of AgNPs with sodium citrate and AiiA was taken in the range of 4,000–500 cm^−1^ on Thermo Scientific Nicolet iS50 FT-IR spectrophotometer (RC, SAIF, PU, Chandigarh). The changes in various functional groups in the chemical composition of molecule were observed to confirm the adsorption of AiiA over AgNPs. Pychem was used to analyze and superimpose the spectra (Jarvis et al., 2006).

### X- Ray Diffraction (XRD) and Energy Dispersive Spectrum (EDS-SEM) to Determine the Physico-Chemical Properties of AgNPs

The crystalline structure of AgNPs was determined by using XRD. The analysis was conducted by using XPERT-PRO. Monochromatic Cu k_α_ radiation (λ = 1.5406 Å) operated at 45 kV and 40 mA at a 2θ angle pattern was used to process samples. The scanning was done in the region of 10°–90°. The images obtained were compared with the Joint Committee on Powder Diffraction Standards (JCPDS) library to account for the crystalline structure. The average crystalline size of the NP was calculated by using Debye-Scherrer formula d = 0.89λ/βcosθ, where d is the particle size, λ is the wavelength of Cu X-ray radiation (1.5406 Å), β is the full-width at half-maxima (FWHM) of the strongest peak (in radians) of the diffraction pattern and 2θ is the Bragg angle (Zhang et al., 2016). The presence of elemental silver (AgNPs) and nitrogen of AiiA was analyzed by energy dispersive spectroscopy attached with Fe-SEM. Specimens were examined at varying voltages using Hitachi SU8010 FeSEM at SAIF (Sophisticated Analytical Instrumentation Facility), Panjab University, Chandigarh.

### Determination of Minimum Inhibitory Concentration (MIC)

To compare the antibacterial efficiency, MIC of azithromycin, neat AgNPs, and AgNP—AiiA complex was calculated. For this, different concentrations of the solutions (0.07–108.4 μg/mL for azithromycin and 0.45–1 mg/mL for neat AgNPs, and AgNP-AiiA complex) were checked for antibacterial activity against *K. pneumoniae* B5O55 by tube dilution method (Serebryakova et al., 2002). The minimum concentration of these compounds that prevented visible growth of *K. pneumoniae* was noted as its MIC.

### Establishment of Biofilm

To prepare inoculum, *K. pneumoniae* was grown in LB and washed with phosphate buffered saline (PBS, pH 7.4). Its O.D._600_ was adjusted, so as to obtain 1 × 10^8^ cells/ml of *K. pneumoniae*. To prepare biofilm in a 24 well polystyrene plates, equal volumes (500 ul) of bacterial culture and media were added in each well. The plates were lid covered and incubated at 37°C without agitation, for 24 h.

#### Determination of Minimum Biofilm Inhibitory Concentration (MBIC)

After the establishment of biofilm, MBIC of azithromycin, neat AgNPs, and AgNP-AiiA complex was determined (Favre-Bonte et al., 2003). The biofilm was exposed to 100 μL of different dilutions of azithromycin (range 1.87–1098.64 μg/mL), neat AgNPs, and AgNP-AiiA complex ranging from 14.4 to 1643.2 μg/mL in LB. After incubating the plate at 37°C for 24 h, the supernatant from each well was carefully transferred to a fresh microtiter plate and turbidity of the contents was measured by taking the absorbance at 600 nm on microplate reader (Biorad, USA). The biofilm MIC was defined as the minimal concentration of compounds at which no visible bacterial growth was observed.

#### Estimation of Cell Surface Hydrophobicity (CSH) by Microbial Adhesion to Hydrocarbon (MATH) Assay

The effect of neat AiiA and AgNPs-AiiA complex on the CSH of *Klebsiella* was estimated by MATH assay. Bacteria were initially grown in luria broth at 37°C for 24 h, harvested by centrifugation at 8,000 rpm for 5 min at 25°C, re-suspended in sterile distilled water, and adjusted to an optical density (OD_600nm_) of 0.2 ± 0.03. The AgNPs-AiiA complex (14.4 μg/mL), AiiA protein (50 μg/mL), and toluene (1 ml) were added to 2 ml of the cell suspension (*A*_600_ = 0.2 ± 0.03). After vortexing, cell suspensions were left for incubation at room temperature overnight, and the OD of the aqueous phase was obtained (*A*_600_). The hydrophobicity index (HI) of *Klebsiella* was calculated as HI = 100 (*E* × 100/*E*^0^) (Favre-Bonte et al., 2003). The results were expressed in the form of proportion of cells which were excluded from the aqueous phase. Here *E*^0^ is the initial optical density of the cell suspension and *E* is the final optical density of the aqueous phase after its separation from the toluene phase.

#### Quantification of Total Exo-Polysachharide (EPS)

To calculate the EPS produced by K. pneumoniae, its biofilm was grown as mentioned in section Establishment of biofilm. After 24 h, the biofilm forming cells were washed and re-suspended in sterile distilled water. Total polysaccharide content was measured by using the phenol-sulphuric acid assay (PSA). For this, 5 ml of 0.5% phenol and 2.5 ml of concentrated H_2_SO_4_ were added to the biofilm cells and left at room temperature for 30 min in a glass test tube. Samples were centrifuged (6,000 g for 15 min), and O.D. of the supernatant was measured spectrophotometrically at 485 nm. For blank, sample was replaced by water and treated in a similar manner. The total carbohydrate was expressed in mg/mL and glucose was used as reference to prepare the standard curve.

#### Assessment of Metabolic Activity of *Klebsiella pneumoniae* in Biofilm by Using XTT Reduction Assay

The effect of treatment with neat AgNPs and AgNP-AiiA complex on the metabolic activity of bacterial cells during the establishment of biofilm was evaluated by using XTT [2,3-bis(2-methyloxy-4-nitro-5-sulfophenyl)-2H-tetrazolium-5-carboxanilide] reduction assay. It measures the colored formazan derivative formed due to the reduction of tetrazolium salts by active bacterial cells, by a calorimetric method. AgNPs-AiiA (14.4 μg/ml) and AiiA protein (14.4 μg/ml) were added to the biofilms in a 24-well polystyrene plate containing LB and 2% (w/v) glucose. Following overnight incubation, the biofilms were washed with PBS (pH7.8) and 14 μl XTT-menadione solution (12.5:1, vol/vol) was added to each wells. After 3 h incubation in dark at 37°C, change in the color of the solution was measured spectrophotometrically by obtaining the absorbance of solution at 450 nm (Fu et al., 2014).

#### AHL Degrading Activity of the AgNPs—AiiA Complex

The 24 h. biofilm of *K. pneumoniae* was treated with AgNP-AiiA complex. After 24 h, the amount of AHL (Acyl homoserine lactone) was determined. The change in the amount of AHLs present in the biofilm was calculated to determine the catalytic efficiency of the complex. Quorum sensing signal molecules (AHLs) produced by *K. pneumoniae* were extracted from the culture supernatant by the method given by Mukesh et al. (2017). Extracted quorum sensing signal molecules (AHLs) were quantified in the culture supernatant on the basis of its β-galactosidase activity. The culture of bio-reporter *E. coli* MG4 was diluted 1:1 in Z-buffer and assayed for β-galactosidase activity by using O-nitrophenyl-D-galactopyranoside (ONPG) as a substrate (Ahamed et al., 2010).

#### Antibacterial Activity of AgNPs-AiiA Complex on 24 h Biofilm

After treating the 24 h. biofilm of *K. pneumoniae* with AgNPs-AiiA complex, viability of the cells was determined by staining with propidium iodide. The dead cells were stained in red color as determined by FACS Canto II system (BD Biosciences) and analyzed with FACS Diva software. The percentage of dead bacterial cells in each case were compared, relating to the efficiency of each compound in inhibiting the biofilm of *K. pneumoniae*.

### Estimation of Silver Mediated Reactive Oxygen Species (ROS) Generation

*K. pneumoniae* (Panktonic form) was exposed to neat AgNPs (14.4 μg/ml) and AgNP-AiiA complex (14.4 μg/ml) to evaluate the difference in intracellular ROS generation after treatment. After exposure, the bacterial cells were recovered, washed twice and resuspended in PBS (7.2 pH) at a concentration of 2 million cells/ml. 2′-7′dichlorofluorescin diacetate (DCFH-DA) was added to 1 ml of bacterial sample and incubated at 37°C for 1 h (Chhibber et al., 2017). The fluorescence signal at 488 nm/525 nm was measured to determine the intracellular ROS production in the exposed *K. pneumoniae* cells.

### Cell Cytotoxicity Testing

Macrophages were taken out from BALB/C mice and cultured by following the protocol given by Singla et al. (2016) and Mitiku and Yilma (2018). For MTT assay macrophage cells were cultured in 50 ml flask containing Dulbecco Modified Eagle Medium (DMEM), 10% FBS at 37°C with 5% CO_2_. Once ~90% confluency was reached, they were harvested by using 0.05% trypsin/EDTA and counted on hemocytometer upon staining with trypan blue. A concentration of 10^5^ cells/mL was obtained and added onto 96-well plate (i.e., 250 μL/well).

#### Treatment With AgNP—AiiA Complex

At 40–50% confluency (42 h. post-inoculation), the cultivated cells were incubated with neat AgNPs (28.4 μg/mL) and AgNP-AiiA complex (22.4 μg/mL). Six wells were left untreated as control. After 3 h, media was removed and replenished with fresh media.

#### Evaluation of Cell Viability by MTT Assay

MTT assay was performed 24 h after treatment. MTT solution (1 mg/ml) was prepared in PBS and filtered through a filter (0.2 μm). Except cell-free wells, 20 μl MTT was added in each well. The plate was incubated for 3 h at 37°C and supplemented with 5% CO_2_. The optical density (OD) of each well was determined by using a plate reader at a test and reference wavelength of 570 and 630 nm, respectively.

Viable cells reduce MTT (3-(4,5-dimethylthiazol-2-yl)-2,5-diphenyltetrazolium bromide) to form formazan crystals. This property of bacterial cells was exploited in this assay. After 24 h incubation, 10 μL of MTT solution was added into each well. The plate was centrifuged (400 g/10 min.) after 4 h of incubation and supernatant was removed. The intra-cellularly stored formazan was dissolved with 300 μL of the solubizing solution after incubation for 8 h at room temperature. The absorbance was determined at 570 nm by using plate reading spectrophotometer (Biorad).

### *In vivo* Toxicity Study of AgNP and AgNP-AiiA

To check the *in vivo* toxicity of neat AgNPs and AgNP-AiiA, female Balb/c mice were injected intravenously with 50 μL of 28.4 μg AgNP and 22.4 μg AgNP-AiiA complex. 48 h after being injected with the final dose of AgNP and AgNP-AiiA, all the mouse were sacrificed by cervical dislocation and organs (Liver, Spleen, Lungs and Kidney) were collected for pathological analysis.

#### Histopathological Evaluation

The collected organs were fixed in 10% formaldehyde solution and then embedded in paraffin. Following suitable processing, organ sections were stained with haematoxylin and eosin (H&E staining). The sections of kidney, liver, spleen, and lung from respective mouse were observed under light microscopy at 40 and 100X magnification. These sections were compared for any pathological changes implicated upon treatment with neat AgNP and AgNP-AiiA.

## Results

### Optimization of Quorum Quenching Activity of *Aiia* in Different Physical Conditions

The enzyme cleaves acyl homoserine lactone molecules, by reversible hydrolysis of ester bond inside the lactone ring. Though it was efficient in cleaving almost all the variants of AHL. Yet, it was found to be most active against C8-HSL at pH 7.0, 37°C and on addition of 10 mM Zn^2+^ (Additional File 1).

**Figure 1:**
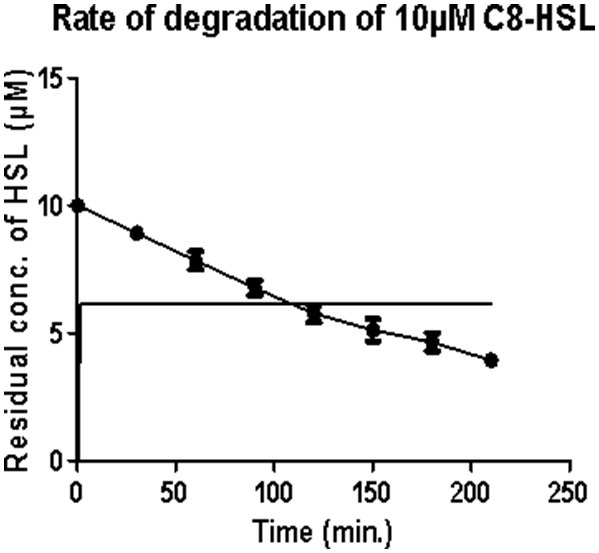
Estimation of residual concentration of homoserine lactones (HSL) to determine the time dependent activity of AiiA. The concentration of C8-HSL was calculated in μM. Each value on the graph represent three independent events in duplicates.

Under optimum conditions, the initial rate of reaction (upto 120 min.) was 35.82 nM/min/100 μg, which then decreased to 18.21 nM/min/100 μg (Figure 1). After 5 h, no zone of purple color was visible indicating the complete degradation of C8 HSL.

### Synthesis of AgNPs

**Figure 2:**
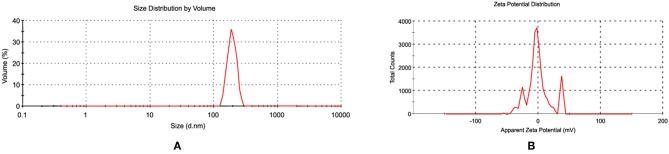
Images showing **(A)** size and **(B)** zeta potential of AgNPs when seen through dynamic light scattering (DLS). NPs (22.4 nm) were prepared in ethanol so as to control their size.

Since the antibacterial activity and toxicity of AgNP depends upon its size, so particles were prepared by varying the concentrations of silver nitrate solution, sodium citrate and AiiA. NPs of 22.4 nm were obtained when 0.001 mM solution of silver nitrate was mixed dropwise with AiiA (100 μg, 100 μL/min) (Figure 2).

The synthesis of NPs was confirmed by aggregating it with sodium chloride (0.017M-2 M, 500 μl). The NPs showed complete aggregation when 300 μl of 0.15 M Nacl was added to an equal volume of 1 mg NPs indicating toward the amount of Nacl needed to neutralize the slight negative charge on AgNPs (Figure 2).

**Figure 3:**
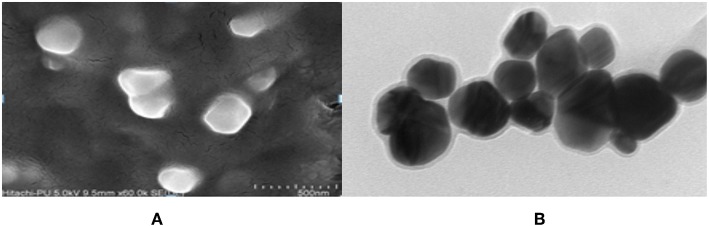
FeSEM images of AgNPs at magnification of **(A)** 2 μm, showing the round shape of NPs. **(B)** proved the round shape of NPs through transmission electron microscopy (TEM) at the scale of 20 nm and magnification of 400000 X. A coating around AgNPs is clearly visible indicating toward the surface modifications by AiiA.

The FeSEM image of AgNPs is shown in Figure 3A. The surface morphology showed even shape, spherical nature, and smooth surface. This was confirmed by TEM analysis as shown in Figure 3B. Figure 3B clearly shows the coating on the surface of AgNPs. This confirms the reduction of silver nitrate to AgNPs by AiiA and adsorption of the latter on the surface.

### Stability Study of AgNPs

**Figure 4:**
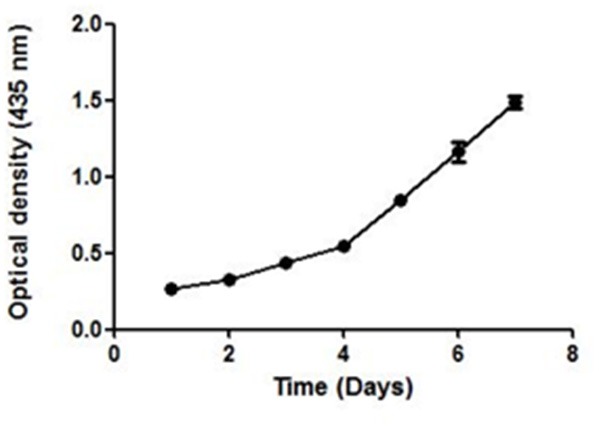
The UV absorbance of AgNPs at 435 nm after an interval of 24 h for 7 days. A continuous increase in the absorbance of nano-particles indicate toward its stability.

UV–Vis spectroscopy is the simplest and an important technique to confirm the formation of NPs. The absorbance spectrum of sample was obtained in the range of 200–800 nm, using a UV–Vis spectrometer (Shimadzu-UV 1800). Maximum absorbance was seen at 435 nm. The growth and nucleation of the NPs was time dependent as measured by taking the optical density at 435 nm after every 24 h, for 7 days. The optical density increased linearly till the 3rd day and thereafter an exponential growth was observed (Figure 4).

A continuous increase in the absorbance indicated toward the nucleation of NPs, thus confirming the formation and maturation of NPs. It resulted in a significant change in the color of NPs with time (Additional File 2). They were stable in the temperature range of 25–40°C as no significant change in their size was seen on varying the temperature (Additional File 3).

### FTIR to Confirm Adsorption of Protein

In order to check the adsorption of AiiA over AgNP, Fourier transform infrared spectroscopy (FTIR) spectroscopy in the range of 500–4,000 cm^−1^ was performed before and after the adsorption of protein. Characteristic peaks at 1566.17, 1388.7071, and 991.09 cm^−1^ indicated the formation of AgNPs.

**Figure 5:**
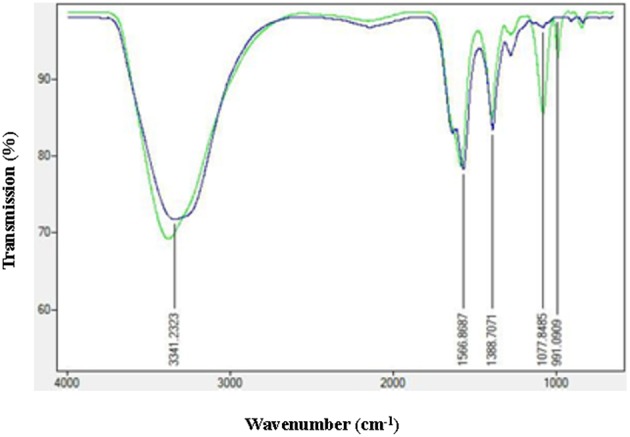
FTIR spectra of AgNPs before and after adsorption with AiiA. Presence of conspicuous peaks at 1,077 nm^−1^ and 991 nm^−1^ indicate toward the interaction between AiiA and AgNP.

Figure 5, shows strong bondings relative to hydrogen bonding (3378.3535 cm^−1^) and amine (-N-C = O) (1,581 cm^−1^), respectively. These bondings might possibly be the reason for efficient adsorption of AiiA and formation of a protein corona around AgNPs.

### XRD and EDS-SEM of AgNPs

**Figure 6:**
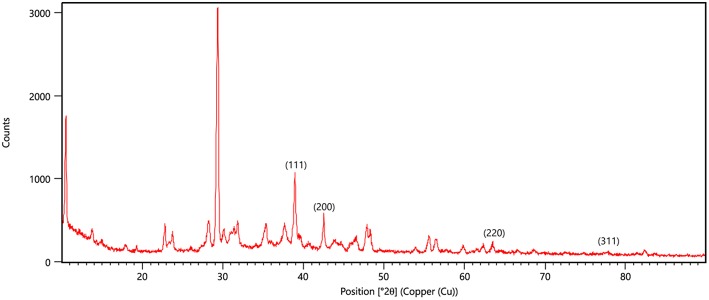
XRD pattern of silver nanoparticles (AgNPs) formed by bombarding with monochromatic Cu radiation. The pattern proves the crystalline nature of nanoparticles with face centered crystal lattice as depicted by the plane of unit cell.

X-ray diffraction peaks were observed at 2θ = 38.890°, 44.057°, 64.371°, and 76.381° (Figure 6). The peaks correspond to hkl values of (111), (200), (220), and (311) of the face-centered cubic (fcc) structure of metallic silver, respectively (standard JCPDS card No. 04-0783 or 87-0597). By estimating the width of Braggs reflection (111), the average crystalline size of the AgNPs was found to be 20.44 nm (Additional File 4).

**Figure 7:**
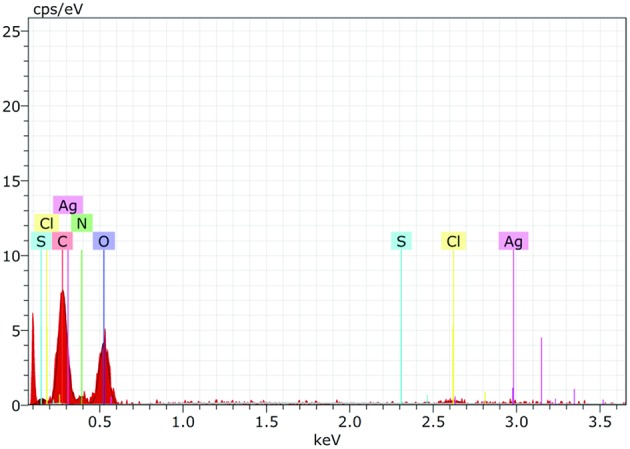
The Energy Dispersive Spectroscopy (EDS) confirmed the presence of elemental silver and AiiA (Protein) inside the reaction mixture. The presence of wide peak of silver around 0.3 keV strongly indicated toward the reduction of silver nitrate to silver nanoparticles (AgNPs).

EDS spectrum reveals strong signals in the silver region and confirms the formation of AgNPs (Figure 7). Metallic silver nanocrystals generally show typical optical absorption peak approximately at 3 KeV due to surface plasmon resonance (Kedi et al., 2018). Silver was the major constituent element along with carbon and oxygen.

Presence of Carbon, Oxygen, Nitrogen, and sulfur confirmed the presence of protein (AiiA). The wide peak of silver indicated the reduction of silver nitrate to AgNPs. The EDS reading proved the presence of required phase of silver (Ag) in the sample.

### Establishment of Biofilm

The 24 h. biofilm was grown and bacterial counts were taken on each day. To establish the biofilm of *K. pneumoniae*, an inoculum containing 10^8^ cfu/ml of *K. pneumoniae* B5O55 was taken.

**Figure 8:**
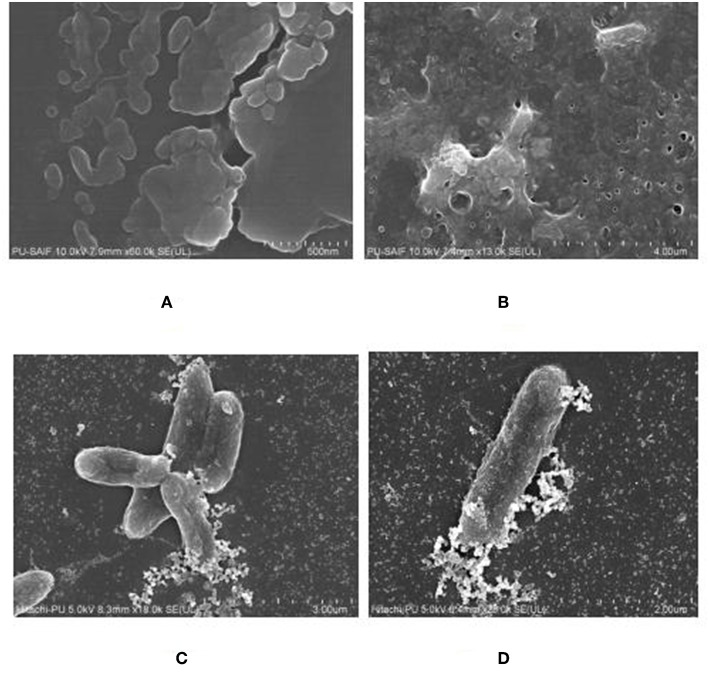
FeSEM images of 24 h. biofilm of *K. pneumoanie* B5O55 showing **(A)** characteristic cocobacilli shape of bacterial cells and **(B)** profuse secretion of extracellular matrix (ECM) by the bacteria during biofilm formation. Water channels are clearly visible as pores in ECM. **(C)** and **(D)** The interaction between nanoparticles and bacterial cells could be seen with a visible decrease in the presence of polysachharide inside biofilm milieu.

The biofilm was grown on polystyrene coverslip and observed by using Fe SEM, to confirm the formation of a mature biofilm characterized by formation of water channels and profuse exo-polysaccharide (EPS), as shown in Figures 8A,B. The interaction between the cells of *K. pneumoniae* and silver nano-particles could been observed from Fe SEM as shown in Figures 8C,D.

### Determination of MIC and MBIC

To calculate the activity of NPs against planktonic cells of *K. pneumoniae* MIC was calculated. After incubating the bacteria with azithromycin (0.07 μg/ml- 108.4. μg/ml) for 24 h, an MIC of 1.87 μg/ml was obtained. Neat AgNPs and AiiA-AgNP complex were added in varying concentrations ranging between 0.45 and 1 mg/ml and both inhibited the growth of *K. pneumoniae* at 14.4 ± 1.27 μg/ml. So this concentration of the additives was used in subsequent experiments. After establishing the biofilm of *K. pneumoniae*, its MBIC was calculated. It was seen that azithromycin inhibited the 24h biofilm at 31.2 μg/ml whereas neat AgNPs and AgNP-AiiA complex inhibited the biofilm at 28.4 μg/mL and 22.4 μg/mL, respectively.

### Hydrophobicity and Metabolism Assays

Bacterial adhesion is a major determinant of biofilm formation as colonization is the first step in any infectious process mediated via formation of biofilm. The hydrophobicity indices of *K. pneumoniae* plummeted sharply upon treatment with AgNPs-AiiA complex as compared to untreated and neat AgNPs treated bacterial cells.

**Figure 9:**
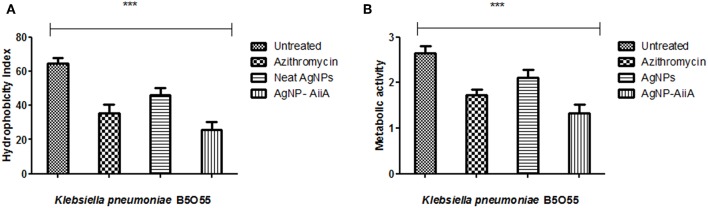
Bar graph showing **(A)** the reduction in hydrophobicity index of K. pneumoniae upon treatment with sub-optimal concentration of azithromycin, neat AgNPs and AgNP-AiiA complex. One way ANOVA proved the result of events as highly significant (*P* < 0.001) **(B)** the reduction in the metabolic activity of K. pneumonaie upon administration of sub-optimal concentration of azithromycin, neat AgNPs and AgNP-AiiA complex (*P*-value 0.0023, ***p < 0.001).

The maximum reduction in the hydrophobicity index caused by AgNP-AiiA complex indicated toward a marked reduction in the number of bacteria, binding to the polystyrene surface which led to the inhibition of biofilm formation (Figure 9A). Further, an XTT assay also proved that treatment with AgNP-AiiA complex reduced the metabolic activity of biofilm more efficiently than treatment with AgNPs alone and untreated samples (Figure 9B).

### Quantification of EPS Production

**Figure 10:**
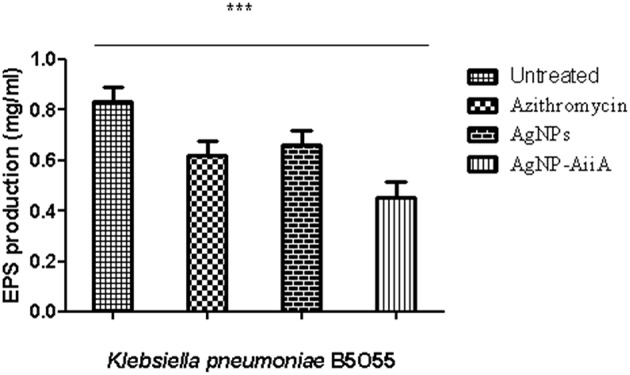
The bar graphs depicts the comparison between production of EPS by *K. pneumoniae* upon treatment with sub-optimal concentrations of azithromycin, neat AgNPs and AgNP- AiiA complex. To compare all groups one way ANOVA was performed and all the events were found to be significant (*P*-value- 0.0115, *p* < 0.05), ***highly significant.

The estimation of synthesis of EPS by *K. pneumoniae* in the presence of Ag NPs-AiiA was done to evaluate the permeability of therapeutic molecules in biofilm (Figure 10).

The reduction in EPS synthesis was greater in the presence of azithromycin than neat AgNPs. However, the application of combination therapy reduced EPS production by 50.48 ± 12.31%. The reduction in EPS production would have greatly affected the viscosity of ECM in biofilm and hence allowing the penetration of therapeutic molecules inside the core of biofilm.

### Viability Assay

To check the viability of bacterial cells present inside biofilm milieu before and after treatment with AiiA (50 μg), AgNPs (14.4 μg/ml), AgNPs-AiiA complex (14.4 μg/ml), and Azithromycin (1.87 μg/ml), they were stained with propidium iodide and killed cells were counted by flow cytometer.

**Figure 11:**
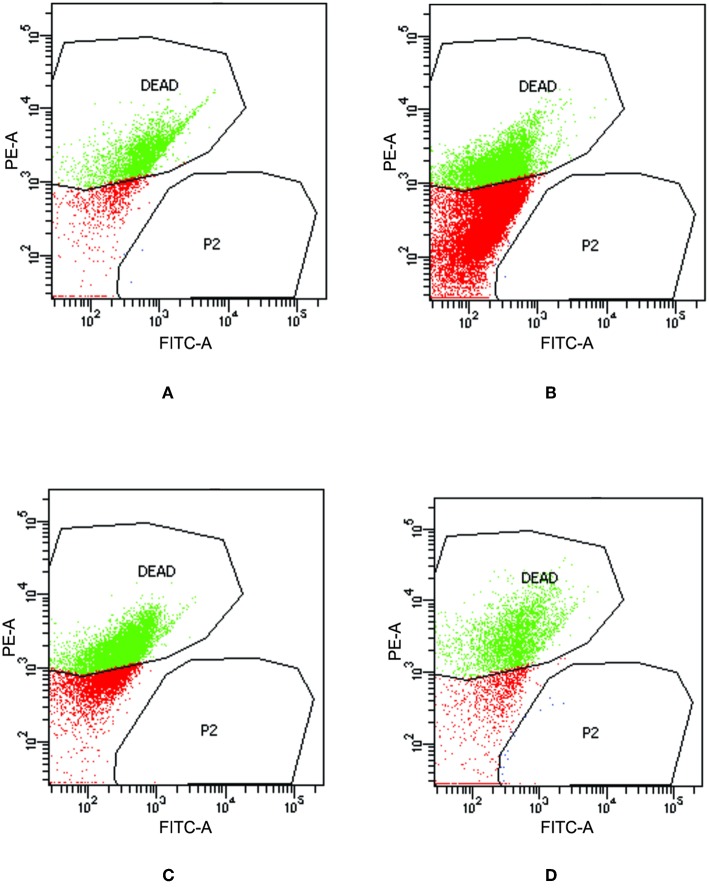
Flow cytometry data showing the viability of *K. pneumoniae* upon treatment with **(A)** AiiA **(B)** Neat AgNPs (14.4 μg/mL) **(C)** Azithromycin (1.87 μg/mL) and **(D)** AgNP- AiiA complex (14.4 μg/mL).

Upon treating biofilm with AiiA, 22.6 ± 3.4 % cells of *K. pneumoniae* were killed (Figure 11A) which increased to 51.9 ± 13.5 % (Figure 11B), 58.4 ± 9.3 % (Figure 11C), and 69.5 ± 11.8% (Figure 11D) cells, respectively, on addition of neat AgNP, azithromycin, and AgNP-AiiA complex. The results indicated that though azithromycin was more efficient than AiiA and AgNPs in killing the biofilm cells of *K. pneumoniae*, however effect of the combination of antivirulent and antibacterial therapy i.e., AgNP-AiiA complex was superior to all other therapies.

### AHL Degradation in Biofilm Setting

The quantitative estimation of AHL from the biofilm of *K. pneumoniae* was done by colorimetric method aided by using a bioreporter strain of *E. coli* MG4 for detecting AHLs. The amount of AHL in biofilm was found to be 491.6 ± 36.2 MU, which decreased by 83.4% upon treatment with AiiA after 24 h to 91.7 ± 5.7 MU. The amount of AHL reduced by 69.14% to 152.39 ± 23.56 MU upon treatment with AgNPs—AiiA complex (14.4 μg/mL), reflecting toward the efficiency of both therapies in quenching quorum sensing signal (AHL).

### Effect of Silver Mediated ROS Generation

To examine the oxidative damage induced by exposure of AgNPs and AgNP- AiiA complex on bacteria, DCFH-DA staining assay was performed.

**Figure 12:**
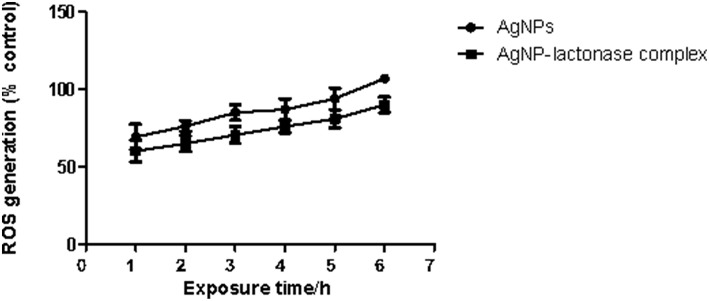
Intracellular ROS generation in *K. pneumoniae* upon time dependent exposure to neat AgNPs and AgNP- AiiA complex. *P*-value < 0.001 (highly significant) (vs. control) was observed for every value of ROS generation.

As shown in Figure 12, exposure to both the additives induced an intracellular ROS generation in a time dependent manner. The induction of oxidative stress was more pronounced in the presence of neat AgNPs as compared to AgNPs-AiiA complex. This might have happened due to less effective concentration of AgNPs in complex as compared to neat formulation.

### *In vitro* Biocompatibility of Silver Nano-Particles

An *in vitro* model was used to evaluate the safety in the use of neat AgNPs and AgNP-AiiA complex. The incubation of AgNPs in different concentrations with primary macrophage cell line was done and analysis of cellular viability (MTT reduction assay) was performed. The viability of untreated macrophage cells remained 100 %.

**Figure 13:**
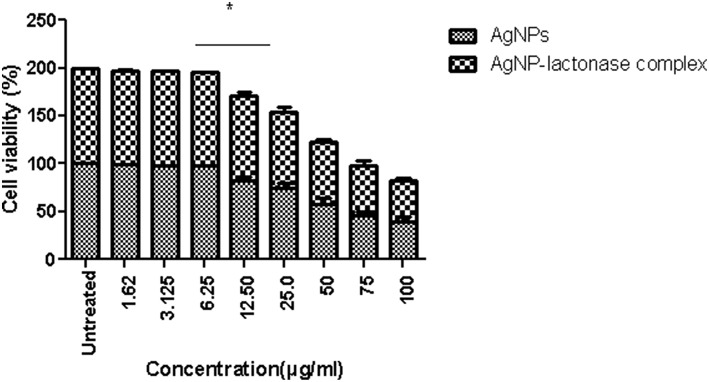
Viability of macrophage cells in presence of varying concentrations (1.62–100 μg/ml) of AgNPs and AgNP-AiiA complex as determined by MTT assay. Bars above the histogram represent standard deviation of mean value of three independent values. Results of the one way ANOVA shows that effect of concentration of AgNPs and AgNP-AiiA complex from 6.25 to 12.50 μg/ml on macrophage cell line is significant (*p* < 0.01, *Significant).

Treatment with neat AgNPs and AgNP-AiiA complex led to a dose dependent effect upon cell viability of primary macrophage cell line. Both the compounds led to a significant decrease in cell viability when used at a concentration of 50 μg/mL, 75 μg/mL, and 100 μg/mL, in comparison with untreated cultures (Figure 13). The results indicated that AgNPs-AiiA were more bio-compatible as compared to neat AgNPs as shown by a higher viability of macrophage cells in the presence of former. No cytotoxic effect at concentrations of 1.5, 3, and 6 μg/mL was observed. The minimum concentration of both compounds that yielded a significant cytotoxic effect was 12.5 μg/mL.

### *In vivo* Cytotoxic Effects of Neat AgNP and AiiA Reduced AgNPs

**Figure 14:**
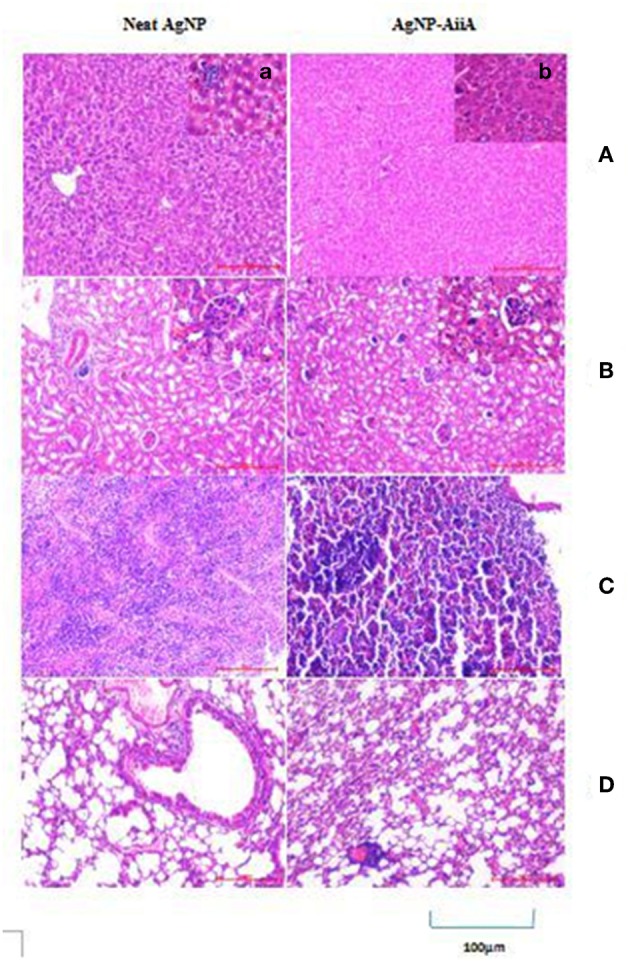
Histological and pathological observations for mice intravenously injected with 14.4 μg neat AgNPs and AgNP-AiiA complex. Each mouse was sacrificed after 48 h of injection and tissue samples of liver, kidney, spleen and lung were collected. **(A)** The representative image of liver. The scale bar in the image represents 100 μm. (a) Enlarged view of Kupffer cells. **(B)** Morphological observation of kidney. (b) Enlarged view of glomerulus. **(C)** Spleen showing red pulp and white pulp. **(D)** Lung showing bronchiole and alveoli.

In the histopathological evaluation, a pigment typical of the presence of AgNPs was observed in organs including spleen and liver. Upon administration of neat AgNPs (28.4 μg/mL), liver was found to be mostly normal with scattered enlarged phagocytic Kupffer cells whereas in case of AgNP-AiiA complex (22.4 μg/mL) it had small cluster of lymphocytes at one edge. In both cases, the changes in pathology of liver were insignificant Figure 14A.

Even in lungs and kidney, no major changes were observed upon i/v injection of neat AgNPs (28.4 μg/mL) and AgNPs-AiiA complex (22.4 μg/mL) as alveoli, bronchioles and glomeruli, respectively, were intact and no change in their shape was observed (Figures 14B,D).

Spleen from mouse injected with 28.4 μg/mL of neat AgNPs had reactive lymphoid cells scattered diffusely both in the white pulp and red pulp indicative of inflammation due to deposition of AgNPs (Figure 14C). On the contrary, upon administration of AgNP-AiiA complex (22.4 μg/mL), small and normal follicles were observed confirming normal pathology of spleen.

## Discussion

NPs have a large surface area as compared to other particulate molecules due to their small size. Thus, a better contact with micro-organisms renders them inhibitory against infectious micro-organisms like *E. coli, S. aureus*, and *P. aeruginosa* (Sarkar et al., 2015). However, concern regarding the possible risks to human health associated with usage of AgNPs has been imminent.

In this study, a two pronged strategy to decimate the bacterial cells present inside 24 h biofilm of *K. pneumoniae* was designed. An attempt was made to cleave the AHL molecules produced by them followed by their killing. For this *aiiA* was cloned from *Bacillus* sp. ZA12 into *E. coli* BL21. It was over—expressed by inducing with IPTG and purified by using Ni-NTA chromatography. Purified AiiA was used to reduce Ag ions by adding the former, dropwise, at a rate of 100 μl/ml. It is known that proteins bind to AgNPs either through free amine group or via cysteine residues (Chunga et al., 2008). The adsorption of AiiA over AgNPs was confirmed by FTIR spectroscopy where formation of amide linkage was observed along with hydrogen bonds. The AgNP- AiiA complex retained both antibacterial and antivirulent activities, as confirmed by MIC and AHL levels, respectively. In the present study, complex was found to be relatively more successful in inhibiting biofilm as compared to AiiA and AgNPs alone (69.5 ± 11.8, 22.6 ± 3.4, and 51.9 ± 13.5%, respectively) in sub-optimal concentrations. This proves the greater antibiofilm potential of AgNP-AiiA complex. It is likely that AiiA might have imparted some antivirulent effect by cleaving AHLs that had accumulated in the biofilm milieu (69.14%), which inturn would have resulted in decreased virulence of the bacteria in biofilm. In a previous report, QS has been shown to regulate attachment of bacteria to the surfaces via hydrophobic interactions. So, the cleavage of AHLs by AgNP-AiiA complex might have led to decrease in the hydrophobic index as well as metabolism of *Klebsiella* inside biofilm structure (Figure 9A).

*Klebsiella* is known to produce 79 types of EPS capsule that acts as a means to avoid immune response and contribute toward serum resistance (Vinoj et al., 2015). EPS also increases the tolerance of the organism toward administered drugs. It was observed that AgNPs—AiiA complex was able to inhibit EPS production by *Klebsiella* and it also disturbed the architecture of biofilm. It is proposed that synergistic interactions between AiiA protein and AgNPs that cleaved the QS molecules, also contributed toward inhibition of biofilm formation. Pan et al. (2015) also reported similar results on the biofilm of *Proteus*, as synergistic effect of gold nano-particles and AiiA was responsible for its inhibition. These observations prove that AgNP-AiiA was detrimental for both bacteria in both planktonic and biofilm form.

The generation of ROS has previously been shown to contribute toward Ag NP-triggered toxicity in bacteria (Shannahan et al., 2015). Over-production of ROS can induce oxidative stress and cells fail to maintain their normal physiological redox regulated functions. This in turn leads to DNA damage, apoptosis, and cancer initiation. There are critical determinants that affect the generation of ROS, including: particle surface, shape, size, and surface charge (Das et al., 2017a). In the present study, it was found that once inside the core of biofilm, AgNP generated ROS and cleaved the cell membrane of Gram- negative bacteria i.e., *K. pneumoniae* as seen through DCHF-DA staining of the bacterial cells. The production of reactive oxygen species induced by addition of AgNP-AiiA complex was less as compared to neat AgNP. This also resulted in decreased death of macrophage cells when incubated with 14.4 μg/ml of the complex.

To compare the *in vitro* cytotoxicity of AgNP-AiiA complex with neat AgNPs, macrophage cells were isolated from BALB/C mice and cultured *in vitro*. A dose dependent effect of the neat AgNPs and AgNP-AiiA complex was observed. Since the effective concentration of AgNPs in AgNP- AiiA complex was less, compared to neat AgNPs, the former was found to be less toxic on primary macrophage cell line as compared to neat AgNPs. The cells adhered to the surface, which further indicated that AgNPs-AiiA had no toxicity on the macrophages. The results are consistent with a previous investigation where AgNPs demonstrated a linear reduction (relative to dose) in viability of both rat lung epithelial (RLE) and rat aortic endothelial (RAEC) cell types at 3 and 6 h after exposure. The formation of protein corona on AgNPs was found to reduce cytotoxicity on both RLE and RAEC at a concentration of 50 μg/ml in case of Human Serum Albumin (HSA) and BSA. In addition, RAEC exposed to AgNPs (25 μg/ml)with High-density lipoprotein (HDL) showed less cytotoxicity at 3 and 6 h (Yan et al., 2018). A reduced cytotoxicity might be due to the altered release kinetics of silver ions induced by enhanced stability of AgNPs upon coating with AiiA.

Upon biofunctionalization, the cytotoxic effect of NPs depends upon its stability (Travan et al., 2009), properties of corona around the NP (Stolle et al., 2005), and interactions between NP and protein (Chen et al., 2017). To prevent aggregation of Ag-NPs, coating is one way to stabilize the NPs by electrostatic and electrosteric repulsions. So, bioactivity of Ag-NPs can be significantly altered upon coating with proteins (Travan et al., 2009). Usually the processes involved in toxicity induction involves ROS generation. However, the method and extent of Ag-NPs toxicity varies based on the coating materials. Proteins can form two types of corona around NPs: soft corona and hard corona. Hard corona does not allow silver ions to penetrate through it and diffuse freely. On the other hand, soft corona allows the penetration of silver ions with some restraint. In this study, ROS was generated upon treatment of *K. pneumoniae* with neat AgNPs, and AgNP-AiiA complex. This confirms the formation of soft corona of AiiA around AgNPs which might have allowed silver ions to move outside the complex. This justifies the antibacterial activity of the complex. However, a reduced oxidative stress in case of treatment with AgNP-AiiA complex also rationalized its reduced cytotoxicity as compared to neat AiiA. Once safety in the use of complex against macrophage cell line was ensured, neat AgNPs, and AgNP-AiiA complex was injected intra-venously inside Balb/C mice and It's *in vivo* safety was ascertained. Upon histopathological evaluation of spleen, liver, lungs, and kidney, spleen from mouse injected with neat AgNPs (28.4 μg/mL) showed reactive lymphoid cells both in white pulp and red pulp indicative of deposition of AgNPs. On the contrary, normal follicles were observed upon treatment with AgNP-AiiA (22.4 μg/mL). This might have been possible due to slow release of silver ions from the complex due to formation of soft corona.

## Conclusion

In the present study, AgNPs coated with AiiA isolated from *Bacillus* sp. ZA12 were prepared and characterized by UV-visible spectra, DLS, FTIR and Fe SEM. AgNPs-AiiA complex was found to be antibacterial and had antibiofilm properties. The inhibition of virulence factors including EPS production, hydrophobicity index, and metabolic activity of *Klebsiella* at sub-optimal concentration was found, thus supporting the case for its antibiofilm property. The results of toxicity studies revealed no changes in the morphology of macrophage cell when treated with AgNPs-AiiA. The outcome of this study might result in development of potential biomaterials against nosocomial pneumonia, burn wound and cystic fibrosis caused by *K. pneumonaie*.

## Ethics Statement

The experimental protocols were performed with prior approval from Institutional Animal Ethics Committee of Panjab University, Chandigarh, India (Approval ID: IAEC/156). All the experiments concerning animals was complied with the guidelines of Committee for the Purpose of Control and Supervision of Experiments on Animals (CPCSEA), Government of India.

## Author Contributions

SC conceived the idea. KG performed all the experiments and wrote the manuscript.

### Conflict of Interest Statement

The authors declare that the research was conducted in the absence of any commercial or financial relationships that could be construed as a potential conflict of interest.

## Supplementary Material

Additional File 1 Histogram depicting the activity of AiiA at different (a) pH (4–8) and (b) temperature (25–50°C). The activity of AiiA has been calculated in nM/min/100 μg). Each value represents the mean of three independent evens performed in duplicates.

Additional File 2 The image shows the visual change in the color of silver nanoparticles upon incubation at room temperature after 7 days. It confirms the nucleation of nanoparticles.

Additional File 3 The table shows the change in size of nanoparticles upon subjection to varying temperature.

Additional File 4 The table shows the calculations required to estimate the average crystalline size of AgNPs reflected by the braggs angle obtained through X-Ray diffraction analysis.
