# Supplementary figures and images for: Biofunctionalization of Silver Nanoparticles With Lactonase Leads to Altered Antimicrobial and Cytotoxic Properties
Source: Front Mol Biosci. 2019 Aug 6;6:63. doi: 10.3389/fmolb.2019.00063 (PMC6691173; doi:10.3389/fmolb.2019.00063)

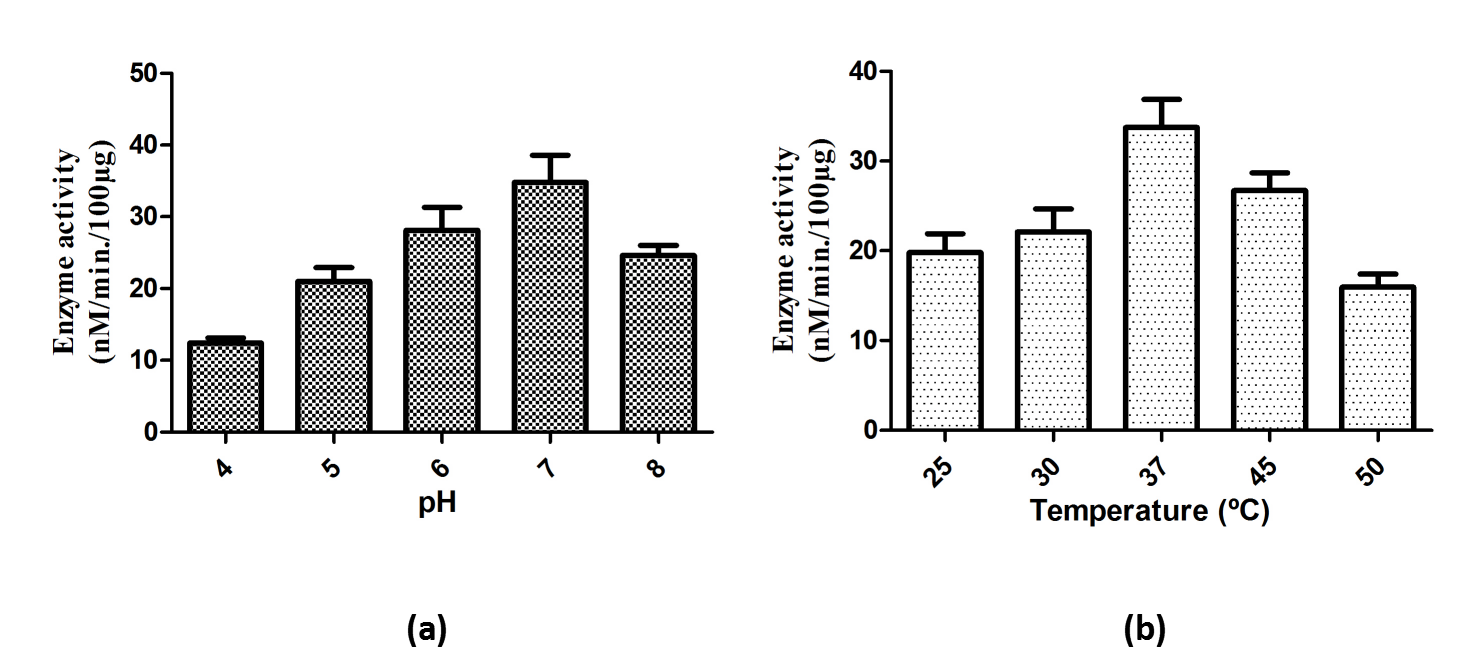

Supplement: Additional File 1 — Histogram depicting the activity of AiiA at different (a) pH (4–8) and (b) temperature (25–50°C). The activity of AiiA has been calculated in nM/min/100 μg). Each value represents the mean of three independent evens performed in duplicates. [file Image_1.tif]

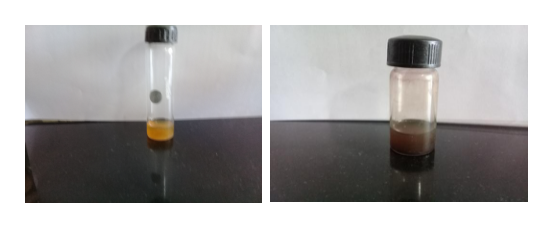

Supplement: Additional File 2 — The image shows the visual change in the color of silver nanoparticles upon incubation at room temperature after 7 days. It confirms the nucleation of nanoparticles. [file Image_2.TIF]

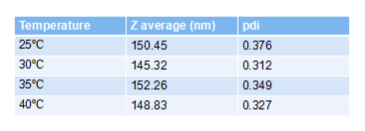

Supplement: Additional File 3 — The table shows the change in size of nanoparticles upon subjection to varying temperature. [file Table_1.DOCX]

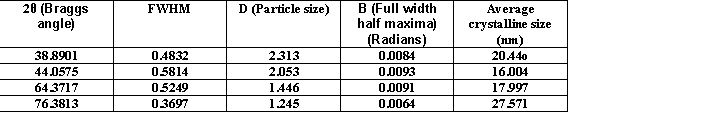

Supplement: Additional File 4 — The table shows the calculations required to estimate the average crystalline size of AgNPs reflected by the braggs angle obtained through X-Ray diffraction analysis. [file Image_3.TIF]
